# Mistletoe lectin is not the only cytotoxic component in fermented preparations of *Viscum album *from white fir (*Abies pectinata*)

**DOI:** 10.1186/1472-6882-7-14

**Published:** 2007-05-10

**Authors:** Jenny Eggenschwiler, Leopold von Balthazar, Bianca Stritt, Doreen Pruntsch, Mac Ramos, Konrad Urech, Lukas Rist, A Paula Simões-Wüst, Angelika Viviani

**Affiliations:** 1 University of Applied Sciences, Einsiedlerstr. 29b, P.O. Box 335, CH-8820 Waedenswil, Switzerland; 2 University of Applied Sciences, Campus Furtwangen, Robert-Gerwig-Platz 1, 78120 Furtwangen, Germany; 3 University of Applied Sciences, Carl-Zeiss-Promenade 2, Postfach 10 03 14, 07703 Jena, Germany; 4 Clinical Research Weleda AG, CH-4144 Arlesheim, Switzerland; 5 Hiscia Research Institute, CH-4144 Arlesheim, Switzerland; 6 Paracelsus-Spital, Research Department, Bergstr. 16, CH-8805 Richterswil, Switzerland

## Abstract

**Background:**

Preparations of mistletoe (*Viscum album*) are the form of cancer treatment that is most frequently used in the complementary medicine. Previous work has shown that these preparations are able to exert cytotoxic effects on carcinoma cells, the extent of which might be influenced by the host tree species and by the content of mistletoe lectin.

**Methods:**

Using colorimetric assays, we have now compared the cytotoxic effects of *Viscum album *preparations (VAPs) obtained from mistletoe growing on oak (*Quercus robur *and *Q. petraea*, VAP-Qu), apple tree (*Malus domestica*,, VAP-M), pine (*Pinus sylvestris*, VAP-P) or white fir (*Abies pectinata*, VAP-A), on the *in vitro *growth of breast and bladder carcinoma cell lines. While MFM-223, KPL-1, MCF-7 and HCC-1937 were the breast carcinoma cell lines chosen, the panel of tested bladder carcinoma cells comprised the T-24, TCC-SUP, UM-UC-3 and J-82 cell lines.

**Results:**

Each of the VAPs inhibited cell growth, but the extent of this inhibition differed with the preparation and with the cell line. The concentrations of VAP-Qu, VAP-M and VAP-A which led to a 50 % reduction of cell growth (IC_50_) varied between 0.6 and 0.03 mg/ml. Higher concentrations of VAP-P were required to obtain a comparable effect. Purified mistletoe lectin I (MLI) led to an inhibition of breast carcinoma cell growth at concentrations lower than those of VAPs, but the sensitivity towards purified MLI did not parallel that towards VAPs. Bladder carcinoma cells were in most cases more sensitive to VAPs treatment than breast carcinoma cells. The total mistletoe lectin content was very high in VAP-Qu (54 ng/mg extract), intermediate in VAP-M (25 ng/mg extract), and very low in VAP-P (1.3 ng/mg extract) and in VAP-A (1 ng/mg extract). As to be expected from the low content of mistletoe lectin, VAP-P led to relatively weak cytotoxic effects. Most remarkably, however, the lectin-poor VAP-A revealed a cytotoxic effect comparable to, or even stronger than, that of the lectin-rich VAP-Qu, on all tested bladder and breast carcinoma cell lines.

**Conclusion:**

The results suggest the existence of cytotoxic components other than mistletoe lectin in VAP-A and reveal an unexpected potential of this preparation for the treatment of breast and bladder cancer.

## Background

Breast cancer ranks as the most frequently diagnosed form of malignant disease and the second most relevant cause of cancer-related death in women living in Europe and North America [1]. The progression of breast cancer can vary considerably since this disease comprises a wide range of malignancies that differ in invasiveness, in prognosis and in the molecular characteristics of the tumor cells. In spite of recent improvements in hormonal therapies and in the use of adjuvant cytotoxic therapies, the reduction in the overall mortality rate has been rather modest and approximately 40 % of breast cancer patients will eventually succumb to their disease [1].

In the United States of America, bladder cancer is the sixth most common malignant disease and the ninth leading cause of cancer-related deaths [2]. The majority (72 %) of newly diagnosed bladder cancers concerns men, women being responsible for only approximately 28 % of the cases. The patients often (75%) suffer from superficial bladder cancer, which is confined to the mucosa and translates into a relatively high survival rate [2]. Patients with unresectable or metastatic disease, however, have low long-term survival prospects in spite of chemotherapy treatment [2]. In general, the survival rates tend to be better in men.

Given that the conventional therapies often lead to only partial success, a relatively high proportion of cancer patients have been trying treatments originating from complementary and alternative medicine. In the case of breast cancer, a recent clinical study involving patients from 11 European countries revealed that some form of complementary/alternative treatments had been used in 45 % of the cases [3]. *Viscum album *preparations (VAPs) are the most commonly used form of complementary/alternative cancer therapy. These preparations are often used in the adjuvant setting, together with standard chemo- or radiotherapy [3]. Post-operative treatment with a mistletoe extract in combination with standard treatment has been shown to improve quality of life and relapse-free intervals in breast cancer patients [4,5]. Furthermore, some patients could better stand aggressive chemotherapies if receiving VAPs at the same time [5]. For updated and critical reviews of the clinical studies concerning the use of mistletoe extracts in cancer therapy see [6,7].

*In vitro *experiments with cell lines and with primary cultures have shown that the various VAPs can be cytotoxic to a variety of carcinoma cells, either through the activation of the apoptotic cascade, or by leading to necrosis [8-11]. Furthermore, these extracts have also been shown to possess immunomodulatory and anti-angiogenic properties [12-16]. Preparations originating from mistletoe bushes that grow in different host trees possess distinct compositions and result in cytotoxic effects of varying magnitude [10]. Furthermore, very recent work has shown that these preparations influence the gene expression profile of breast carcinoma cells differentially [17]. The cytotoxic effect of VAPs is likely to be at least partially caused by mistletoe lectin I (MLI), and the recombinant counterpart of this protein has been shown to be a powerful agent that is able to reduce cell proliferation and to induce apoptosis in the 0.1–1 ng/ml concentration range [18,19]. Nevertheless, the *in vitro *toxicity of VAPs does not always correlate with their lectin content, suggesting that other components [20] might also play a role [10,17].

In the present study, the effects of VAP-A, VAP-M, VAP-P and VAP-Qu obtained from mistletoe growing on Abies (white fir), Malus (apple tree), Pinus (pine), and Quercus (oak), respectively, on the *in vitro *growth of several breast and bladder carcinoma cell lines were studied.

## Methods

### Materials

VAP-Qu (Charge Nr. 41011), VAP-M (Charge Nr. 30611), VAP-P (Charge Nr. 40511) and VAP-A (Charge Nr. 41111), as well as the isolated MLI were provided by Hiscia AG (Arlesheim, CH); total mistletoe lectin concentrations of the preparations were determined by the manufacturer. MLI was isolated by affinity chromatography on lactose-Sepharose using (NH4)_2_SO_4_-precipitates of extracts from *V. album ssp. album *grown on apple trees (elution by 0.1 M lactose); MLI was not contaminated by MLII and MLIII as controlled by SDS-gel-electrophoresis; the concentration of MLI was determined using an ELLA-test. 3-(4, 5-dimethylthiazol-2-yl)-2, 5-diphenyltetrazolium bromide) (MTT) and fetal calf serum (FCS) were purchased from Sigma Chemical Co. Aldrich (Switzerland), dimethyl sulfoxide (DMSO) and trypan blue from Fluka (Switzerland), Dulbecco's Modified Eagle's Medium high glucose (DMEM), RPMI 1640, Dulbecco's phosphate buffered saline (PBS) and trypsin-EDTA solution (0.25% trypsin/0.05% EDTA) from AMIMED BioConcept (Switzerland). The cell lines MFM-223 (ACC 422), KPL-1 (ACC 317), MCF-7 (ACC 115), HCC-1937 (ACC 513), T-24 (ACC 376), TCC-SUP (ACC 377) were supplied by DSMZ (Deutsche Sammlung von Mikroorganismen und Zellkulturen GmbH, Germany), whereas the cell lines UM-UC-3 (CRL-1749) and J-82 (HTB-1) were purchased at ATCC (American Type Culture Collection, Rockville, MD, USA).

### Cell culture

All cell lines were cultured as recommended by the suppliers and kept at 37°C in a humidified atmosphere with 5% CO_2_. The cells, all of which exhibited an adherent morphology, were sub-cultured twice a week by washing with PBS and digesting the monolayer with trypsin/EDTA at 37°C for 5 min.

### Cell growth assay

Cells were seeded at the density of 10^5 ^cells/ml in 100 μl/well (or 10,000 cells/well), in a 96-well polycarbonate plate and incubated for 24 hours at 37°C and 5 % CO_2_. Thereafter, the cells were exposed to different concentrations of appropriate dilutions of VAPs, purified MLI or blank medium (control) for 48 h, in a total volume of 200 μl/well. The previously described MTT-colorimetric assay was then used to estimate cell growth [9]. In brief, 20 μl of a 5 mg/ml MTT solution were added to each well. After 4 h at 37°C, 200 μl DMSO (Fluka, Switzerland) were added to each well, the plates were gently shaken for 10 min, and finally 25 μl Sørensen's buffer (0.1 M glycine, 0.1 M NaCl, pH 10.5) were added and the absorbance was measured at 570 nm on a microplate reader (MRX, Dynatech Laboratories) against 620 nm. The absorbance value of cells treated with culture medium and vehicle control was taken as 100 % cell growth. The toxic effect of each dilution of the mistletoe preparations was calculated by taking the OD mean value of sixteen treatment-wells relative to the OD mean value of twenty-four control-wells, unless mentioned in the context. Each experiment was repeated at least twice. Data are shown as mean ± S.D. Note that in this assay cell growth reflects the total mitochondrial reducing ability of the cell populations, which itself is strongly influenced by the corresponding proliferation rate (in a positive way) and by the occurrence of cell death (in a negative way).

### IC_50 _determination

The data used for determining the concentrations of VAPs and of MLI which resulted in a 50% inhibition of cell growth (IC_50_) were collected in colorimetric MTT-assays performed as described above. The assays included the following concentrations of mistletoe preparations: 4, 2, 1, 0.5, 0.1, 0.01 and 0.001 mg/ml. In the case of the assays with MLI, 1000, 100, 10 and 1 ng/ml were tested. Computer-aided curve fitting and statistical analyses were performed using the commercially available software package IGOR Pro (Version 4.0.8.0, Wavemetrics, Inc., 10200 SW Nimbus, G-7, Portland, OR 97223, USA). The curves were modeled using the following sigmoid fit for a logarithmic scale: y = A2 + (A1-A2)/(1 + (x/x0)^p). The confidence level was set at 68 %.

## Results

### Effect of VAPs on the in vitro growth of the KPL-1, MCF-7, HCC-1937 and MFM-223 breast carcinoma cell lines

**Figure 1:**
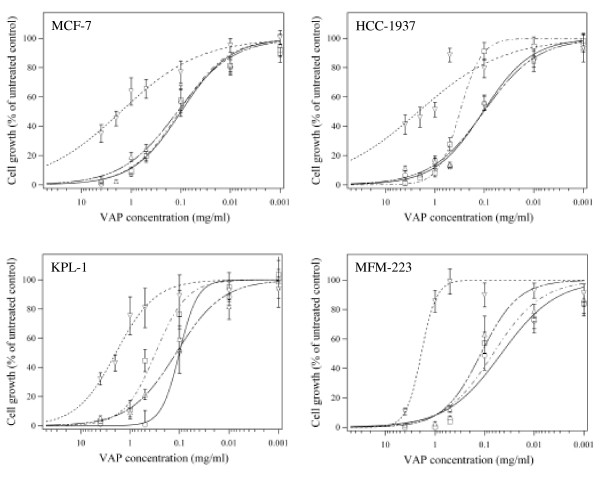
Effects of VAPs on breast carcinoma cell growth. MCF-7, HCC-1937, KPL-1 and MFM-223 breast carcinoma cells were treated with different concentrations of VAP-Qu (○), VAP-M (△), VAP-P (▽) or VAP-A (□), during 48 hours. Thereafter cell growth was determined using the MTT-colorimetric assay and the data were calculated as described under Methods. The growth of cells kept under comparable experimental conditions but in the absence of VAPs and of MLI (untreated control) was taken as 100 %. Each experiment was repeated at least twice. Data are shown as mean ± S.D.

Fig. 1 shows that each of the tested VAPs was able to inhibit the *in vitro *growth of the MCF-7, HCC-1937, KPL-1 and MFM-223 breast carcinoma cells. However, the extent of this inhibition varied markedly with the preparation and with the cell line. While MCF-7 cells reacted similarly to VAP-A, VAP-M and VAP-Qu, the remaining cell lines exhibited some preference for one of these preparations. Confirming previous observations [17], VAP-P exhibited the weakest effect on all breast carcinoma cells. To better compare the effects of the various VAPs on the different breast carcinoma cells, the corresponding IC_50 _values after 48 hours of treatment were calculated and are depicted in Table 1. The data show that MFM-223 cells were most sensitive to VAP-A (IC_50 _of 0.05 mg/ml), HCC-1937 cells to VAP-M (IC_50 _of 0.10 mg/ml) and VAP-Qu (IC_50 _of 0.11 mg/ml), and KPL-1 cells to VAP-Qu (IC_50 _value of 0.10 mg/ml).

**Table 1:** Concentrations of various VAPs and MLI that are able to inhibit the growth of MFM-223, KPL-1, HCC-1937 and MCF-7 breast carcinoma cell lines by 50 % (IC_50 _values).

	MFM-223	KPL-1	MCF-7	HCC-1937
VAP-Qu	0.05 ± 0.017	0.10 ± 0.013	0.09 ± 0.024	0.11 ± 0.017
VAP-M	0.12 ± 0.034	0. 12 ± 0.022	0.12 ± 0.020	0.10 ± 0.020
VAP-P	1.89 ± 0.300	1.94 ± 0.278	1.61 ± 0.248	2.14 ± 0.989
VAP-A	0.07 ± 0.028	0.31 ± 0.059	0.10 ± 0.022	0.31 ± 0.019
MLI	38 ± 3.4	141 ± 29.0	410 ± 59.0	320 ± 73.2

The cells were treated for 48 hours either with a VAP or with isolated MLI. The values were calculated as described under Material and Methods. The concentrations of mistletoe preparations are expressed as mg/ml; those of MLI as ng/ml.

### Effect of purified MLI on the in vitro growth of the breast carcinoma cell lines KPL-1, MCF-7, HCC-1937 and MFM-223

Since MLI has often been considered to be responsible for the cytotoxic effect of mistletoe extracts, the inhibitory effect of isolated MLI on breast carcinoma cell growth was studied. Purification and characterization of ML I was performed as described under Methods. The IC_50 _values of MLI calculated for the various breast carcinoma cell lines are depicted in Table 1. They are 300–3000 times lower than those obtained with VAPs. Furthermore, the sensitivity profile of the different breast carcinoma cell lines towards MLI did not parallel the sensitivity profile towards VAPs. MFM-223 cells were by far more sensitive to MLI than all the other cell lines, whereas KPL-1, MCF-7 and HCC-1937 were almost equally sensitive to VAP-M. Treatment with MLI affected breast cancer cell morphology, being associated with a more round form, smaller size, formation of cell-aggregates and detachment from the surface (data not shown).

### Effect of VAPs on the in vitro growth of the bladder carcinoma cell lines T-24, TCC-SUP, UM-UC-3 and J-82

**Figure 2:**
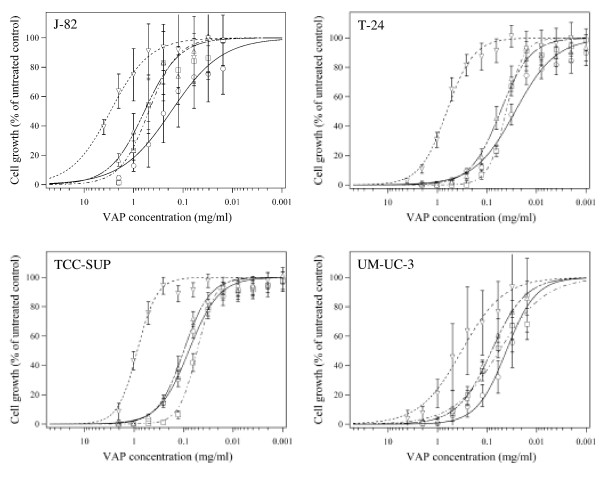
Effects of VAPs on bladder carcinoma cell growth. J-82, T-24, TCC-SUP and UM-UC-3 bladder carcinoma cells were treated with different concentrations of VAP-Qu (○), VAP-M (△), VAP-P (▽) or VAP-A (□), during 48 hours. Cell growth determination and data presentation as in legend to Fig. 1.

Fig. 2 shows that VAPs were able to inhibit the in vitro growth of each of the tested bladder carcinoma cell lines. The IC_50 _values obtained with the various bladder carcinoma cells were determined after 48 hours of treatment and are depicted in Table 2. Each of the four bladder carcinoma cell lines tested turned out to be highly sensitive to the various mistletoe preparations, especially if one compares the results to those obtained with the above mentioned breast carcinoma cell lines (Table 1). With the exception of VAP-P, which again had the weakest effect on cell growth, the IC_50 _values of the VAPs ranged between 0.03 and 0.60 mg/ml (Table 2). We point out that the lectin-poor VAP-A and the lectin-rich VAP-Qu had comparatively strong effects on all bladder cell lines.

**Table 2:** Concentrations of various VAPs that are able to inhibit the growth of bladder carcinoma cells by 50 % (IC_50 _values).

	T-24	TCC-SUP	UM-UC-3	J-82
VAP-Qu	0.03 ± 0.003	0.08 ± 0.005	0.04 ± 0.003	0.17 ± 0.025
VAP-M	0.05 ± 0.005	0.10 ± 0.006	0.08 ± 0.003	0.60 ± 0.127
VAP-P	0.65 ± 0.047	0.86 ± 0.070	0.33 ± 0.039	2.95 ± 0.605
VAP-A	0.04 ± 0.002	0.05 ± 0.003	0.06 ± 0.006	0.49 ± 0.077

The cells were incubated in the presence of the corresponding mistletoe preparations for 48 hours and the values were calculated as described under Material and Methods. All concentrations are expressed as mg/ml.

### Lectin content and strength of cytotoxicity

The mistletoe lectin contents of the VAPs used in the experiments described above are shown in Table 3 (M. Werner, Hiscia AG, personal communication). All VAPs had 20 mg of fermented extracts per ml and the lectin content was expressed relative to the extract weight, to allow a better comparison with preparations standardized at other concentrations. The mistletoe lectin concentration was the highest in VAP-Qu with 53.5 ng/mg, approximately half this concentration in VAP-M and 40–50 times lower in VAP-A and VAP-P (Table 3).

**Table 3:** Mistletoe lectin concentration of the VAPs used in the experiments.

Mistletoe preparation	Lectin (ng/mg extract)
VAP-Qu	54± 1.45
VAP-A	1.0 ± 0.02
VAP-M	25 ± 0.03
VAP-P	1.3 ± 0.30

Data are shown as average ± S.D. of four measurements.

## Discussion

Our observations corroborate previous data showing that VAPs were able to inhibit the *in vitro *growth of breast [9] and bladder [11] carcinoma cells and that the extent of this inhibitory effect varied with the mistletoe host tree [10,17]. There are differences in the responsiveness of the cell lines which were chosen to represent either breast or bladder cancer. The four bladder carcinoma cell lines turned out to be generally more sensitive to mistletoe preparations than the four breast carcinoma cell lines. These data strongly support clinical investigations on the possible use of VAPs in the context of bladder cancer. It is worth mentioning that the superficial forms of this malignancy are good candidates for instillation (intravesical therapy), which is likely to constitute an almost optimal delivery system for VAPs at high local concentrations.

The IC_50 _values of VAP-Qu, VAP-M and VAP-A for the various breast and bladder carcinoma cell lines were lower than those obtained with VAP-P, indicating that this latter preparation has the weakest effect on viability/proliferation of carcinoma cells among the four VAPs tested. The IC_50 _value of each preparation varied to some extent with the cell line, which suggests that in the clinical context it might be necessary to select the most effective preparation to be used in each breast or bladder cancer patient. This selection will probably be more complex than a mere determination of the mistletoe lectin level of the various extracts, because: a) the sensitivity profile towards isolated MLI did not parallel that towards VAPs, and b) the mistletoe lectin content of the various extracts was not always proportional to the corresponding cytotoxic effect.

The hypothesis that the mistletoe lectin content of a preparation determines the magnitude of its inhibitory effect on carcinoma cell growth seems to be confirmed by the data obtained with VAP-P. This preparation, which had a very low level of mistletoe lectin (1.3 ng/mg extract, Table 3), revealed the weakest cytotoxic effect. Strikingly, however, VAP-A had even slightly less mistletoe lectin than VAP-P (1.0 ng/mg extract, Table 3), but the strongest or second strongest cytotoxic effect of all four VAPs on MFM-223, MCF-7, T-24, TCC-SUP, UM-UC-3 and J-82 carcinoma cells. These results suggest that other components might (also) play a role in the observed cytotoxic effect of VAPs, and in particular of VAP-A. Viscotoxins, which are usually present in the mistletoe preparations, are among the possible candidates to have caused this additional effect on cell growth [21,22]. This group of toxins includes several isoforms whose levels vary among the VAPs, VAP-P exhibiting the lowest levels of viscotoxins A1, A2 and A3 among the tested extracts [22]. Interestingly, VAP-A, which revealed a strong cytotoxic effect on the various cell lines, but whose mistletoe lectin content was very low (approximately fifty times less than that of VAP-Qu, for instance), is the preparation with the highest level of viscotoxin A3 [22]. It is therefore conceivable that viscotoxin A3 might play a major role in the cytotoxic effect of VAP-A. Viscotoxin A3 is known to interact strongly with biomembranes due to its pronounced hydrophobic character [21].

Should the effect of VAP-A be mainly mistletoe lectin-independent and those of the other preparations mainly mistletoe lectin-dependent, one would expect that VAP-A would induce a different set of intracellular processes than VAP-Qu or VAP-M. In line with these expectations, very recent data on the gene expression profile in breast carcinoma cells indicate that VAP-A induces molecular changes on the cell-cell adhesion and cytoskeleton pathways, while VAP-Qu and VAP-M mainly affect the immune defense and stress response genes [17].

## Conclusion

Our work suggests that the differences among the various VAPs might have been so far underestimated and that the assumption that their cytotoxic effect relies exclusively on the mistletoe lectin content is likely to be a simplification. It is conceivable that VAP-A opens new possibilities to explore the full potential of *Viscum album *in breast and in bladder cancer therapy. To our knowledge, and in contrast to the situation in breast cancer, the data concerning the clinical use of mistletoe preparations on bladder cancer, even though very promising, are scarce [23]. The potential of mistletoe preparations in bladder cancer therefore deserves clinical investigations.

## Competing interests

Within the last five years AV, LR and APSW have received occasional research funding from the pharmaceutical company Weleda AG, who produces VAP. MR has received salaries from this company. The other authors declare that they have no competing interests.

## Authors' contributions

JE coordinated the practical part of the project and carried out the experiments with the breast carcinoma cell lines, LB performed the data analysis, BS and DP carried out the cytotoxicity assays with the bladder carcinoma cell lines. MR provided scientific support. KU isolated and purified ML I. AV conceived the study and helped to draft the manuscript. APSW drafted and wrote the manuscript with LR. All authors read and approved the final manuscript.

## Pre-publication history

The pre-publication history for this paper can be accessed here:


